# Metoclopramide combined with probiotics improves gastric retention in mechanically ventilated patients following craniocerebral surgery

**DOI:** 10.3389/fnut.2025.1714169

**Published:** 2026-01-09

**Authors:** Yuling An, Baoyu Zhang, Qinqin He, Ziyu Li, Xiaomeng Yi, Huimin Yi, Hui Wang

**Affiliations:** 1 Department of Surgical Intensive Care Unit, The Third Affiliated Hospital of Sun Yat-sen University, Guangzhou, China; 2 Department of Neurosurgery, The Third Affiliated Hospital of Sun Yat-sen University, Guangzhou, China

**Keywords:** craniocerebral surgery, gastric retention, metoclopramide, neurosurgery, probiotics

## Abstract

**Objectives:**

This study aimed to evaluate the effect of the co-administration of metoclopramide and probiotics on enteral feeding tolerance in mechanically ventilated patients after cranial surgery.

**Methods:**

From January 2023 to December 2024, a total of 88 patients presenting with acute brain injury and treated by craniocerebral surgery were screened. Of these, 32 were excluded and 56 patients were enrolled and assigned to either the intervention group (*n* = 32) or the control group (*n* = 24). Enteral nutrition was initiated within 24 h following nasogastric tube placement. The intervention group received a combined regimen of metoclopramide and probiotics for 3–7 days in addition to the standard enteral nutrition protocol. Feeding complications and functional outcomes were compared between the two groups.

**Results:**

Compared to the control group, the intervention group exhibited a statistically significant reduction in total gastric residual volume (GRV) during the first 3 days of gastric tube feeding (103.1 ± 47.8 ml versus 756.3 ± 137.1 ml, *P* < 0.05). The intervention group demonstrated a significant reduction in vomiting and diarrhea incidence within 7 days (3.1% versus 20.8%; 6.3% versus 25%, all *P* < 0.05), higher serum albumin levels at 2 weeks (33.1 ± 1.5 g/L versus 31.8 ± 1.5 g/L, *P* < 0.05), and a shorter hospital stay (17.8 ± 4.1 days versus 23.7 ± 5.1 days, *P* < 0.05). However, the groups did not differ significantly in 3-month postoperative modified Rankin Scale (mRS) scores (4.2 ± 0.8 versus 4.3 ± 1.0, *P* > 0.05).

**Conclusion:**

The co-administration of metoclopramide and probiotics significantly reduced gastrointestinal intolerance such as gastric retention, vomiting, and diarrhea in mechanically ventilated patients following craniocerebral surgery.

## 1 Introduction

Delayed gastric emptying, a condition frequently observed in patients with acute brain injury, is primarily attributed to impaired cerebral function (1). Although adherence to certain clinical guidelines is relatively high, significant discrepancies persist between recommended practices and actual implementation in intensive care units (ICUs), resulting in suboptimal nutritional therapy (2). Notably, gastric retention occurs in over 80% of patients with intracerebral hemorrhage (3). Among those with elevated gastric residual volume, motility agents and small bowel feeding were administered in 58.7% and 14.7% of cases, respectively. Furthermore, nutritional adequacy for energy and protein intake averaged 59% and 60.3% respectively (3). A cross-sectional survey conducted among ICU nurses and clinicians in China between December 2019 and January 2020 demonstrated a positive perception of preventive strategies for feeding intolerance. In patients with severe traumatic brain injury, the commonly utilized measures to mitigate this condition included nasogastric tubes (91.2%), probiotics (79.0%), and prokinetic agents (73.3%) (4).

Delayed gastric emptying can precipitate reflux and pulmonary aspiration, potentially interrupting enteral nutrition administration and hindering the achievement of caloric targets (2). Moreover, the development of pneumonia imposes a substantial metabolic demand, significantly increasing nutritional requirements (5). Furthermore, postoperative nausea and vomiting (PONV) are the most frequent sequelae of craniotomy, occurring in roughly 50% of patients (6, 7). These complications pose a life-threatening risk by inducing dangerous surges in arterial and intracranial pressure (8).

Current clinical strategies for the prevention and management of gastric retention encompass post-pyloric enteral nutrition administration, the utilization of gastroprokinetic agents, positional adjustments, and reduction of feeding rates (5, 9). However, empirical evidence indicates that 38% of patients continue to exhibit feeding intolerance for an average duration of 1.9 ± 1.3 days despite the implementation of these interventions (10). This underscores the critical need for the development of a simple, safe, and efficacious approach to mitigate gastric retention in clinical practice.

This study aimed to explore a simple drug supplementation regimen and ultimately identified the co-administration of metoclopramide and probiotics in the treatment of gastrointestinal intolerance after craniotomy. Both agents have been extensively studied in the field of neurocritical care. Metoclopramide is a D2 receptor antagonist with central dopaminergic activity that enhances gastric motility and is recommended as a rapid prokinetic agent for enteral nutrition support in the intensive care unit (11–13). Comprehensive evidence from multiple systematic reviews and meta-analyses indicates that probiotic supplementation as an adjuvant therapy in neurosurgical patients, including those with traumatic brain injury and after spinal surgery, provides clear benefits, including significantly improved gastrointestinal motility, modulated inflammatory responses, and reduced infection rates (14–17). The interaction between prokinetic drug and probiotics is primarily mediated through modulation of the gut microbiota composition, microbial metabolites, and host immune function (18, 19). By enhancing gastrointestinal motility, metoclopramide may indirectly alter the distribution of intestinal flora, potentially increasing the proportion of potential pathogens such as *Escherichia coli* and *Proteus*. Probiotics containing strains such as *Bifidobacterium* can partially counteract the adverse effects of metoclopramide on the gut microbiota.

This study intended to evaluate the safety and efficacy of this combination therapy with a view to laying the foundation for future quality improvement initiatives in clinical practice.

## 2 Objects and methods

### 2.1 Object

This retrospective cohort study examined patients who underwent enteral nutrition following craniocerebral surgery at our institution between January 2023 and December 2024. The study protocol was reviewed and approved by the Institutional Review Board of The Third Affiliated Hospital of Sun Yat-sen University. Written informed consent was secured from the legal representatives of all participants.

Included in the study were adult patients aged 19–80 years who were required to meet the following criteria: (1) confirmation of acute brain injury via preoperative cranial CT leading to craniocerebral surgery; (2) a Glasgow Coma Scale (GCS) score of 3–13; (3) a requirement for mechanical ventilation of at least 48 h; and (4) initiation of continuous pump-assisted enteral nutrition via a nasogastric tube within 24 h postoperatively in the ICU. Patients were excluded from the study if they met any of the following criteria: they had undergone a repeat craniotomy; presented with severe hepatic or renal dysfunction, pre-existing gastrointestinal disorders, acute spinal cord injuries, or anterior cranial fossa fractures; required therapeutic hypothermia or prone positioning ventilation; or died within 14 days of admission.

### 2.2 Nutritional regimen

A standardized post-craniotomy therapeutic protocol was implemented for all acute brain injury patients admitted to our Surgical Intensive Care Unit (SICU), including dehydration therapy, intracranial pressure reduction, pharmacological management, and prophylactic measures against fever, infection, and stress ulcers (20). Insulin therapy was commenced for persistent hyperglycemia, defined as two consecutive blood glucose measurements exceeding 150 mg/dL.

We implemented a uniform nutritional protocol for both groups; enteral nutrition was initiated within 24 h after nasogastric tube placement. The enteral nutritional suspension (TPF, Nutricia Pharmaceutical Co., Ltd., Wuxi, China) was administered via an enteral feeding system (Baitong, Qingdao Ruicong Medical Equipment Co., Ltd., China) at a controlled temperature of 37–40 °C and a consistent infusion rate of 60–80 mL/h. The caloric and nitrogen intake targets were set at 25 kcal/kg/day and 0.2 g nitrogen/kg/day, respectively. On the first day, 50% of the target caloric intake was delivered via gastric tube, increasing to 60% on the second day and reaching 70%–100% by the third day and thereafter. Gastric residual volume (GRV) was assessed every 6 h and by extracting gastric content with a sytinge. Elevated GRV was defined as two consecutive measurements exceeding 200 mL. If the cumulative GR surpassed 500 mL within a 24-h period or if vomiting occurred, enteral nutrition was suspended for 6 h and subsequently resumed at the previous infusion rate.

Patients in the control group received standard enteral nutrition support without additional interventions. Those in the intervention group received the standard protocol plus a combined regimen of metoclopramide and probiotics for 3–7 days. Metoclopramide was administered intravenously at a dose of 10 mg, two to three times daily. Probiotics (Live Combined Bacillus Subtilis and Enterococcus Faecium Enteric-coated Capsules, Hanmi Pharm. Co., Ltd.) were administered orally, one capsule three times daily.

### 2.3 Assessment of nutritional status and complications

For each included patient, we collected basic information and comorbidities. We also evaluated gastrointestinal intolerance indicators, nutritional indicators, pneumonia, mechanical ventilation time, ICU time, hospitalization time, mortality rate, and modified Rankin Scale (mRS)scores at 90 days.

Nutritional status was evaluated through the measurement of serum albumin and hemoglobin levels. These biomarkers were quantified one day prior to the initiation of enteral feeding and subsequently at 2 weeks post-initiation.

The evaluated complications included gastric retention, pulmonary aspiration, diarrhea and pneumonia. These complications were diagnosed in accordance with the criteria from a previously established study (5). Pulmonary aspiration was characterized by sudden episodes of choking, respiratory distress, or the expulsion of sputum resembling the nutrient solution. Diarrhea was defined as the occurrence of loose or watery stools, accompanied by hyperactive bowel sounds and a frequency of at least three bowel movements per day. Suspected cases of pneumonia underwent bronchoalveolar lavage (BAL) for pathogen culture. A definitive diagnosis of pneumonia required the presence of a new, persistent infiltrate on chest radiograph, plus at least two of the following criteria: (1) body temperature > 38.3 °C; (2) leukocytosis (a ≥ 25% increase from baseline with an absolute count > 10,000 WBC/mm^3^) or leukopenia (a > 25% decrease from baseline with <25 WBC per high-power field). A positive BAL culture, while not mandatory, provided supporting evidence for the diagnosis. All nurses involved in the care of these critically ill patients were well-trained and evaluated for proficiency in applying these diagnostic criteria.

### 2.4 Data and statistical analysis

The primary outcome was defined as the total volume of gastric residual volume (GRV) collected over the 72-h period following the initiation of gastric tube feeding. Secondary outcomes comprised: (1) the incidence of vomiting and diarrhea within 7 days after the intervention; (2) the development or progression of pneumonia, assessed by physicians blinded to treatment allocation; (3) occurrence of new infections within 7 days, based on clinical diagnosis by the attending physician; (4) nutritional indicators (albumin, HGB) at 14 days of feeding and prognostic indicators (mechanical ventilation time, ICU time and length of hospital stay);(5) functional outcomes, measured by the median modified Rankin Scale (mRS) score at 90 days (±14 days) and independently evaluated by two treatment-blinded investigators; and (6) tracheostomy rate and all-cause mortality within 90 ± 14 days.

All statistical analyses were performed using SPSS version 25.0 (IBM Corp., Armonk, NY, USA). The normality of data distribution was evaluated using the Shapiro–Wilk test. Continuous variables were expressed as mean ± standard deviation (SD) and compared using Student’s *t*-test; non-normally distributed data were summarized as median (range) and analyzed with the Mann–Whitney U test. Categorical variables were presented as frequencies (percentages) and compared using the chi-square test or Fisher’s exact test, as appropriate. A two-sided *p*-value < 0.05 was considered statistically significant.

## 3 Results

**FIGURE 1:**
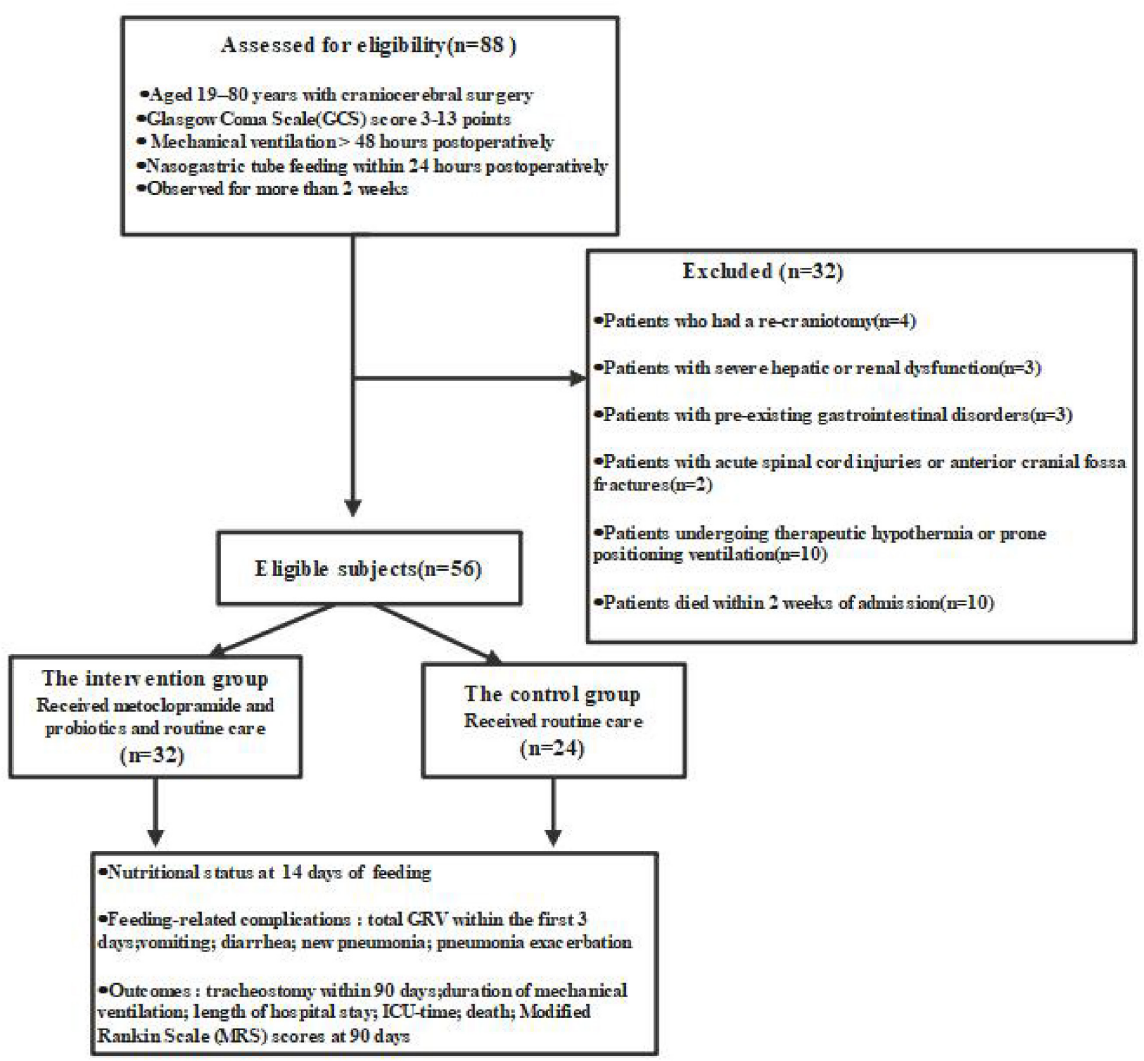
Participant flowchart through the study.

From January 2023 to December 2024, a total of 88 patients presenting with acute brain injury and treated by craniocerebral surgery were screened. Of these, 32 were excluded according to the pre-defined criteria, leaving 56 patients who were ultimately enrolled and assigned to either the intervention group (*n* = 32) or the control group (*n* = 24) as shown in Figure 1.

Baseline demographics and clinical characteristics of participants in both groups as shown in Table 1. Comparative analysis confirmed no significant differences (*P* > 0.05) in all measured covariates, including age, sex, body mass index (BMI), Acute Physiology and Chronic Health Evaluation (APACHE) II score, Glasgow Coma Scale (GCS) score, serum albumin levels, hemoglobin levels, comorbidities, primary disease, mechanical ventilation and pre-enrollment pneumonia status.

**TABLE 1:** Baseline characteristics of both groups (*n* = 56).

Variable	Intervention group (*n* = 32)	Control group (*n* = 24)	*P*-value
Age (years) mean ± SD	61.3 ± 10.4	62.1 ± 10.0	0.79
Gender (female/male), *n*	3/29	5/19	0.23
BMI (kg/m^2^) mean ± SD	24.9 ± 1.6	24.6 ± 1.8	0.64
APACHE II score median (range)	13 (9–17)	13 (9–17)	0.59
GCS score median (range)	9 (6–12)	9 (6–12)	0.74
Albumin before enteral feeding (g/L) mean ± SD	33.1 ± 1.5	32.7 ± 2.1	0.40
Hemoglobin before enteral feeding (g/L) mean ± SD	96.3 ± 11.3	96.1 ± 14.0	0.97
**Comorbidities before hospitalization *N* (%)**			
Hypertension	13 (40.6%)	9 (37.5%)	0.81
Diabetes	3 (9.4%)	2 (8.3%)	0.89
Coronary heart disease	2 (6.3%)	1 (4.2%)	0.73
Heart failure	1 (3.1%)	0	0.38
COPD	2 (6.3%)	1 (4.2%)	0.73
Previous stroke	1 (3.1%)	1 (4.2%)	0.84
Immunocompromized	2 (6.3%)	0	0.21
**Primary disease that led to craniotomy *N* (%)**			
Traumatic brain injury	5 (15.6%)	4 (16.7%)	0.92
Intracerebral hemorrhage	19 (59.4%)	12 (50.0%)	0.48
Subarachnoid hemorrhage	5 (15.6%)	4 (16.7%)	0.92
Cerebral infarction	3 (9.4%)	4 (16.7%)	0.41
Mechanical ventilation (yes) *N* (%)	30 (93.8%)	23 (92.0%)	0.80
Pneumonia (yes) *N* (%)	4 (12.5%)	3 (12.5%)	1.0

BMI, body mass index; APACHE, Acute Physiologic Assessment and Chronic Health Evaluation; GCS, Glasgow Coma Scale; COPD, chronic obstructive pulmonary disease.

**FIGURE 2:**
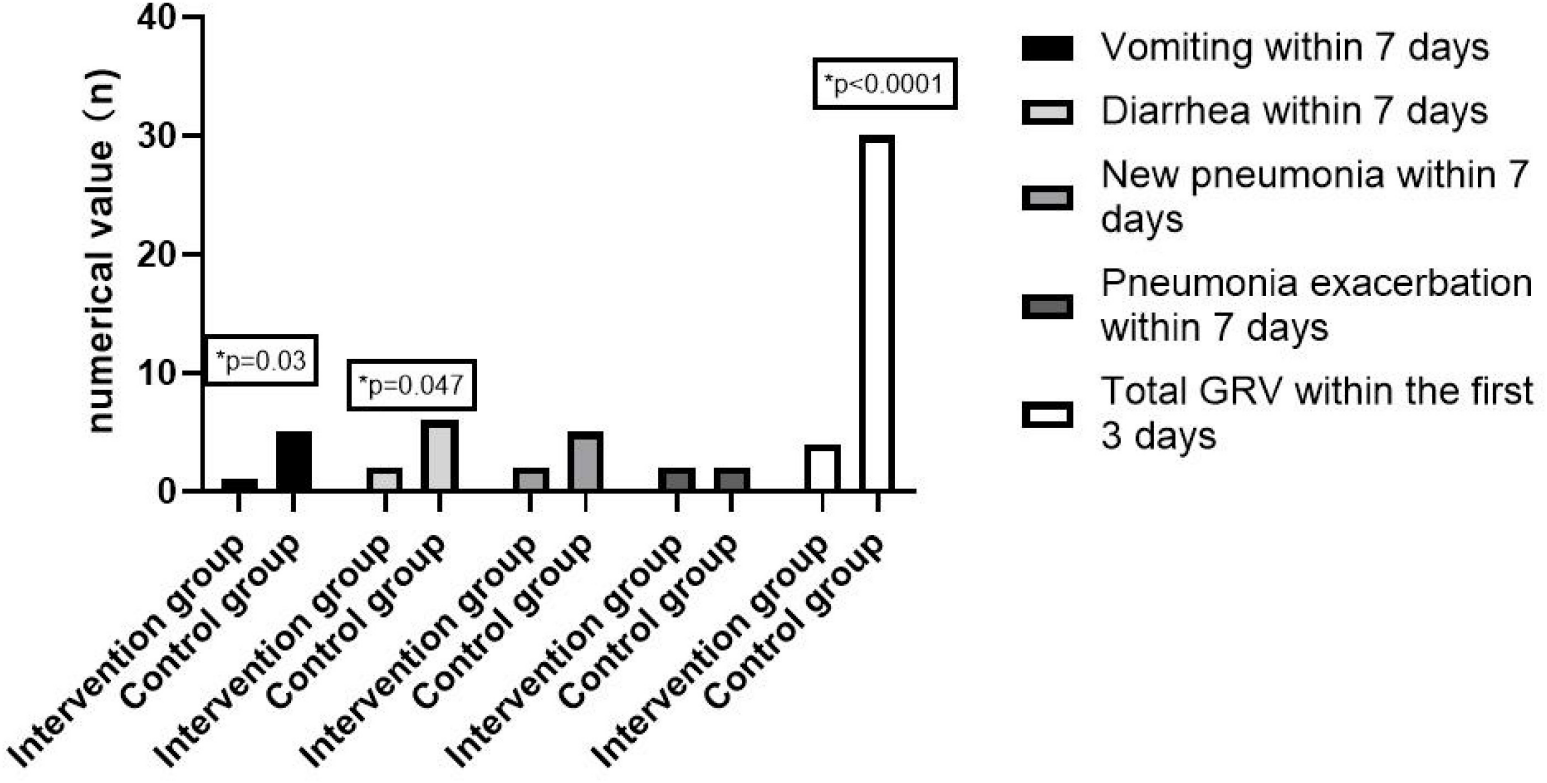
Comparison of feeding-related complications.

Compared to the control group, the intervention group exhibited a marked reduction in total gastric residual volume (GRV) over the first 3 days of gastric tube feeding (*P* < 0.05). The total GRV within 3 days of the start of feeding in the intervention group was 103.1 ± 47.8 ml, while that in the control group was 756.3 ± 137.1 ml. There was a significant difference in the incidence of vomiting and diarrhea between the two groups (*P* < 0.05). The incidence of vomiting in the intervention group was 3.1%, compared to 20.8% in the control group, while the incidence of diarrhea in the intervention group was 6.3%, compared to 25% in the control group. Although not statistically significant, the incidence of new pneumonia within 7 days of feeding was 6.3% in the intervention group compared with 20.8% in the control group, pneumonia exacerbation within 7 days of feeding was 6.3% in the intervention group compared with 8.3% in the control group (shown in Table 2 and Figure 2).

**TABLE 2:** Comparison of feeding-related complications between the two groups.

Variable	Intervention group (*n* = 32)	Control group (*n* = 24)	*P*-value
Total GRV within the first 3 days of feeding (ml) mean ± SD	103.1 ± 47.8	756.3 ± 137.1	<0.0001
Vomiting within 7 days of feeding *N* (%)	1 (3.1%)	5 (20.8%)	0.03
Diarrhea within 7 days of feeding *N* (%)	2 (6.3%)	6 (25%)	0.047
New pneumonia within 7 days of feeding *N* (%)	2 (6.3%)	5 (20.8%)	0.10
Pneumonia exacerbation within 7 days of feeding *N* (%)	2 (6.3%)	2 (8.3%)	0.76

GRV, gastric residual volume.

**FIGURE 3:**
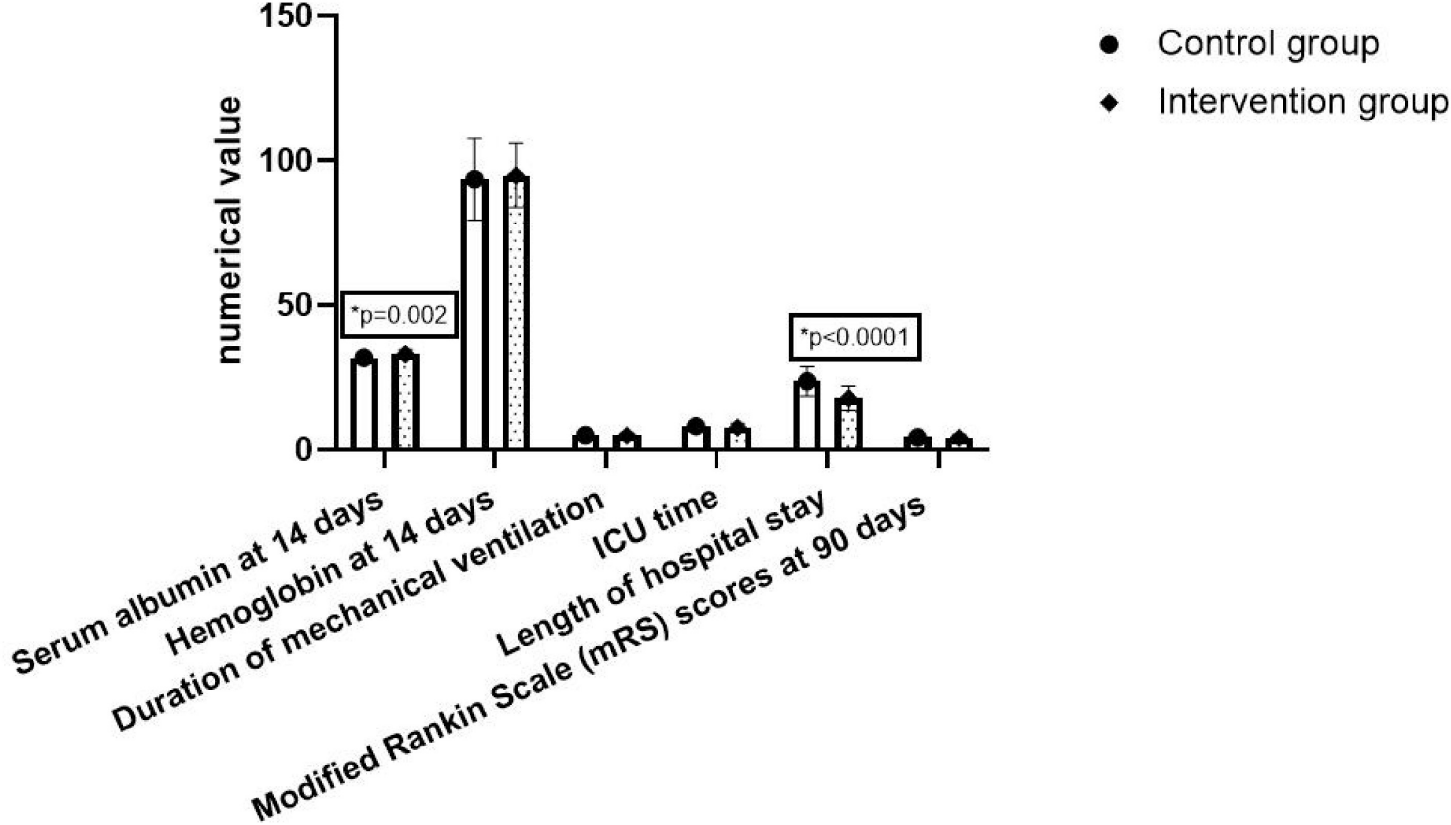
Comparison of outcomes between the two groups.

The serum albumin level of the intervention group at 14 days of feeding was 33.1 ± 1.5 g/L, while the control group was 31.8 ± 1.5 g/L (*P* < 0.05). The length of hospital stay of the intervention group was 17.8 ± 4.1 days, while the length of hospital stay of the control group was 23.7 ± 5.1 days, with a significant statistical difference between the two groups (*P* < 0.05). No statistically significant difference were found in hemoglobin levels at 14 days of feeding, ventilator duration, ICU duration, tracheotomy rate within 90 days, death within 90 days and mRS scores at 90 days between the two groups (shown in Table 3 and Figure 3).

**TABLE 3:** Comparison of outcomes between the two groups.

Variable	Intervention group (*n* = 32)	Control group (*n* = 24)	*P*-value
Serum albumin at 14 days of feeding (g/L) mean ± SD	33.1 ± 1.5	31.8 ± 1.5	0.002
Hemoglobin at 14 days of feeding (g/L) mean ± SD	94.8 ± 11.1	93.5 ± 14.2	0.68
Duration of mechanical ventilation (days) mean ± SD	4.9 ± 1.0	5.1 ± 1.2	0.53
ICU time (days) mean ± SD	7.6 ± 1.5	8.0 ± 1.7	0.44
Length of hospital stay (days) mean ± SD	17.8 ± 4.1	23.7 ± 5.1	<0.0001
Tracheostomy within 90 days *N* (%)	11 (34.4%)	8 (33.3%)	0.94
Death within 90 days (*n*, %)	1 (3.1%)	2 (8.3%)	0.58
Modified Rankin Scale (mRS) scores at 90 days mean ± SD	4.2 ± 0.8	4.3 ± 1.0	0.55

mRS, modified Rankin Scale.

## 4 Discussion

This clinical investigation targeted adults (30–75 years) with acute brain injury following craniotomy, who were admitted to the ICU for mechanical ventilation. It aimed to evaluate the efficacy of pharmacological strategies in mitigating postoperative complications, especially feeding intolerances like gastric retention.

Extensive clinical investigations have established that the co-administration of metoclopramide and dexamethasone in craniotomy anesthesia protocols significantly attenuates the incidence of postoperative nausea and vomiting (PONV) during the initial 24-h postoperative period when compared to dexamethasone monotherapy (17). The randomized phase II MAPS trial (Metoclopramide for Avoiding Pneumonia After Stroke Trial) demonstrated a substantial reduction in pneumonia incidence from 87% to 27% among stroke patients with nasogastric tube placement, though the observed decrease in 30-day mortality from 40% to 27% did not reach statistical significance (18). Conversely, a *post hoc* analysis of the PRECIOUS trial failed to demonstrate that prophylactic metoclopramide, administered over 4 days, conferred any significant benefit in reducing pneumonia risk, improving functional outcomes, or reducing 90-day mortality among elderly stroke patients using nasogastric tubes (19). In the present study, we evaluated the therapeutic efficacy of a combined regimen comprising metoclopramide and probiotics administered over a 3–7 day period in nasogastric tube-fed patients following craniocerebral surgery. The intervention demonstrated notable clinical benefits, including a significant reduction in gastric retention within 3 days of feeding initiation and a decreased incidence of pneumonia progression within the first postoperative week. The intervention group showed numerically lower rates of both new-onset pneumonia (6.3% vs. 20.8%) and pneumonia progression (6.3% vs. 8.3%) compared to the control group. However, these differences were not statistically significant (*p* > 0.05).

Probiotic supplementation has been shown to significantly improve key clinical outcomes in neurosurgical patients, including those with traumatic brain injury or undergoing spinal surgery. Evidence from systematic reviews and meta-analyses confirms its efficacy as an adjuvant therapy, specifically by enhancing gastrointestinal motility, modulating inflammatory responses, and reducing infection rates (14–17). A systematic meta-analysis by Yi et al., which included 18 RCTs (*n* = 1,016 patients) published between 2010 and 2016, demonstrated that for patients with severe traumatic brain injury, early probiotic-supplemented enteral nutrition was associated with significant reductions in infections, mortality, and ICU length of stay (14). However, another systematic review and meta-analysis of seven randomized controlled trials (RCTs) showed no significant effects of probiotics on CRP, IL-6 levels, or ICU length of stay in TBI patients (21). Emerging evidence from a prospective animal study suggests that lactobacilli and other probiotics may confer therapeutic benefits for ischemic stroke by modulating the gut-spleen-brain axis (22). Our preliminary research has found that the intervention group adopting an early combined probiotics treatment plan can reduce the incidence of new pneumonia within 1 week after craniotomy and shorten the length of hospital stay.

Antibiotic-associated diarrhea (AAD) represents a prevalent complication among neurosurgical patients, frequently associated with the administration of surgical antibiotic prophylaxis (SAP) (23, 24). According to a recent systematic review and meta-analysis, adult patients receiving probiotics alongside antibiotics had a 37% lower risk of developing antibiotic-associated diarrhea (AAD) compared to controls (RR = 0.63, 95% CI 0.54–0.73, *p* < 0.00001) (25). Consistent with these findings, our study demonstrated a significant reduction in diarrhea incidence in the intervention group following probiotics treatment.

Probiotics offer a practical therapeutic option in clinical settings due to their wide availability, low cost, and established safety profile. However, a significant knowledge gap persists regarding the neurological or cognitive effects of probiotics supplementation in patients with traumatic brain injury (TBI). Our findings failed to confirm that the co-administration of metoclopramide and probiotics can improve the mRS score at 90 days after craniotomy. Further investigations are warranted to validate the potential therapeutic benefits of probiotics in acute brain injury, optimize strain selection, dosage, timing, and duration of administration, and establish robust evidence for their clinical efficacy (12, 26, 27).

Several limitations of this study warrant consideration. First, one primary limitation is that the relatively small sample size, combined with the retrospective design of the study, may have introduced selection bias. Second, the unavailability of comprehensive data precluded a thorough evaluation of intervention-related adverse effects, particularly QT interval prolongation and extrapyramidal symptoms, including muscle tremors. Third, although all patients were recruited from a single center and followed a standardized feeding protocol, the feeding success rate and protein delivery were lower than in other studies, potentially limiting the generalizability of our findings. Fourth, some patients were transferred or discharged before the removal of nasogastric tubes, precluding the recording of tube duration in all cases. Finally, the limited follow-up period may have hindered the ability to draw more definitive conclusions in this cohort. Critical parameters including the optimal dosage, administration timing, treatment duration, and specific probiotics strains remain to be elucidated.

## 5 Conclusion

The present study revealed that the co-administration of metoclopramide and probiotics significantly improved gastric retention in patients following craniocerebral surgery. Future studies should employ rigorously designed randomized controlled trials with sufficient sample sizes and standardized protocols to validate these preliminary results.

## Data Availability

The original contributions presented in this study are included in this article/supplementary material, further inquiries can be directed to the corresponding authors.
